# Assessment of a training project of English as a media of instruction(EMI) using Kirkpatrick model

**DOI:** 10.1186/s12909-023-04204-5

**Published:** 2023-04-20

**Authors:** Wenlan Zhao, Zixian Liu, Tong Wang, Xiaohan Yin, Yanchun Sun, Xuemei Zhang, Hui Yang

**Affiliations:** 1 Center for Faculty Development, Kunming Medical University, Kunming, China; 2 School of Foreign Languages, Southwestern University of Finance and Economics, Chengdu, China; 3 School of Medicine, Kunming Medical University, Kunming, China; 4 Department of Teaching Management, First Affiliated Hospital of Kunming Medical University, Kunming, China; 5 Institute of Higher Education Research, Kunming Medical University, Kunming, China; 6 Clinical Skill Training Center, Kunming Medical University, Kunming, China

**Keywords:** Kirkpatrick evaluation model, English as a media of instruction (EMI) training, Continue professional development, Medical education

## Abstract

**Background:**

English as a Media of Instruction (EMI) teacher development project is based upon the framework for teacher Continuing Professional Development (CPD) and aims to effectively improve both the confidence and overall capacity of EMI lecturers. Kunming Medical University(KMU) conducted the EMI training project to improve teachers’ competence for MBBS education. This study aimed to assess teachers’ changes following the implementation of this training project, via the Kirkpatrick evaluation model.

**Methods:**

A total of trainees (n = 84) were invited as the research objects. The effects of the EMI training project implemented in KMU were evaluated in terms of the reaction, learning, and behavior dimensions based on the Kirkpatrick model. The self-administered online anonymous questionnaires and observations of participants’ EMI lectures were administered to all participants to collect the data. Furthermore, to understand participants’ perceptions of the management and trainers of the training project, some open-ended questions were required to answer.

**Results:**

Based on 1–3 level of the Kirkpatrick model, all participants were highly satisfied with the EMI training implementation on the reaction level, and expressed positive comments about the management of the training and trainers. On the learning level, participants’ scores on awareness of EMI teaching techniques increased significantly(t = 7.122, P < 0.001)with the training process. Concerning the behavior level, the participant’s confidence as an EMI instructor increased dramatically at end of the whole training(p < 0.001). Moreover, trainees had applied some EMI skills in class and would like to make some commitment to implement learner-centered learning, to do more practice on EMI techniques.

**Conclusion:**

The findings of this study confirm that EMI training has an effective impact on the competence and confidence of participants as EMI instructors at levels 1–3 of the Kirkpatrick evaluation model. This training may be a potentially beneficial effect on the teaching quality of MBBS education.

**Supplementary Information:**

The online version contains supplementary material available at 10.1186/s12909-023-04204-5.

## Background

Amid the internationalization of higher education, Kunming Medical University(KMU) is dedicated to elevating the level of international education effectively and qualified for international students who were studying in English-medium Bachelor of Medicine and Bachelor of Surgery (MBBS) programs admission in 2011. As part of the most vital role in MBBS education, a teacher’s competence in learner-centered teaching and confidence as an EMI instructor in the classroom is necessary to ensure the high quality and effectiveness of teaching. However, lecturers of KMU have a considerable distance to meet the requirements of MBBS education.

The British Council initiate a series of techniques of English as a media of instruction (EMI) teacher development projects based on the framework for teacher Continuing Professional Development (CPD), centered on Academic Teaching Excellence (ATE). It aims to effectively improve the confidence and the overall competence of the lecturers who offer the lessons taught in English, applying the teaching excellence curriculum developed jointly by the British Council and Oxford University. The course was designed to equip the participants with fundamentals of pedagogy and background knowledge of EMI, to develop a series of techniques for making their classrooms more interactive and based on a learner-centered learning(LCL) model, ensuring ‘productive’ upon their return to the class at the end of the project implemented.

However, the conventional didactic Lecture-Based Learning (LBL) method is a much-used learning modality in the medical education field [1], especially in KMU. Although LBL is an effective method for short-term learning to large audiences [2], and is regarded as a teacher-centered educational approach whereby knowledge is transmitted by and from the teacher and passively received by the students [3]. But promoting lifelong learning skills and learner-centered has become a key goal of educators in the twenty-first century worldwide [4]. Meanwhile, medical education is shifting to competency-based education [5], this represents a pedagogical shift from a teacher-centered to a learner-centered focus. However, although instructors have explored actively learner-centered methods that produce lifelong learners in recent years in KMU, there is still a big challenge especially facing MBBS students who are native or near-native speakers of English and came from different cultural contexts and experiences. Therefore, continuing professional development (CPD) of the EMI teaching technique have been served as an imperative step to equip lecturers’ competence with MBBS education in KMU.

To effectively improve teachers’ competence, and to assure positive results of the EMI training project on the quality of MBBS classroom teaching. KMU cooperated with the British Council and conducted three sessions of the EMI training project during 2017–2019. Although, training-specific evaluation is valuable to determine the effectiveness of the training and changes in the knowledge and competencies of the trainees [6], and is important to assess whether the program is tailored to meet the needs and objectives of the program [7]. But this training project has not yet applied an appropriate or systematic approach to analyze and evaluate its effectiveness.

Among the diverse methodologies of educational evaluation [8, 9] Kirkpatrick’s evaluation model was proposed for the first time by Kirkpatrick in the 1960s, has been accepted and used extensively since then, and is a well-recognized standard for evaluating and appraising training programs. [10, 11] It consists of four levels. Reaction(Level 1)measures trainees’ responses to training, including their satisfaction. Learning(Level 2)assesses changes in trainees’ knowledge and skills, indicating the effectiveness of the training. Behavior (Level 3)evaluates changes in trainees’ behavior in the work environment and determines the extent to which knowledge, skills, and attitudes are transferred. Outcome (Level 4)measures the overall impact of training on organizational performance [12]. Furthermore, some studies suggested that the Kirkpatrick model is particularly suitable for evaluation in continuing professional development (CPD) and is adapted for use in higher education and healthcare training programs.[13–15] Thus, It has the most suitable characteristics for the evaluation of EMI programs. This study’s objectives were to evaluate changes in the teaching competence and confidence of trainees on the 1–3 level of the Kirkpatrick model after the EMI training program was implemented at KMU.

## Materials and methods

### EMI training program

**Fig. 1:**
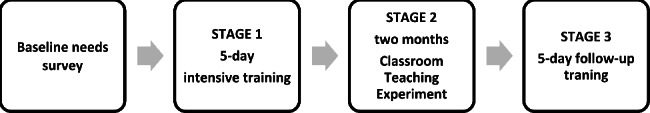
Framework for EMI training program

The EMI training program at KMU mainly embodies a three-stage instructor development program grounded firmly in classroom teaching practices. At the beginning of each session, a baseline survey about the trainees’ basic characteristics was undertaken to identify potential gaps that needed to be addressed in the EMI training. Stage 1 was an intensive 5-day training. Trainees participate in courses covering various aspects of linguistics (language) and pedagogy (teaching). Stage 2 consisted of trainees participating in live classroom experiments. Trainees apply what they have learned to practice and reflect in the live classroom for two months. Stage 3 was a 5-day follow-up training, in which the participants would give EMI lectures/courses to the trainer and other peers to observe. The post-observation analysis and discussion would be organized by the trainer as well. (Fig. 1)

### Participants

#### Sample size and sample selection procedure

This study adopted purposive sampling. The study population participated in EMI training during 2017–2019. We recruited a total of 84 trainees as research objects who came from different departments of KMU.

#### Inclusion criteria

The trainees who took part in all activities of each stage of the EMI training program and were available at the time of the study were invited to participate in this study.

#### Exclusion criteria

The trainees who did not participate in all activities of the training project or trainees with no time to answer the questionnaires were excluded from the sample.

#### Study design and instrument

This study applied the Kirkpatrick evaluation model to evaluate teachers’ changes after EMI programs were implemented in KMU from 2017 to 2019. In terms of the reaction, learning, and behavior dimensions based on the Kirkpatrick model, self-made questionnaires and open-ended questions were used in this study. To assess the reaction level, a feedback questionnaire was conducted at end of the whole training. It consists of two domains that include the following: four closed questions to evaluate the content, value, and quality of the training project and two open-ended questions to measure participants’ perceptions of the management and trainers of the training project. At the learning level, a self-administered online questionnaire (Likert 5-point scale, ranging from “totally unaware, unaware, neither, aware, totally aware” with a corresponding rating of 1 to 5) was used to assess participants’ awareness of 10 learner-centered skills as an EMI instructor at end of stage 1 and 3 respectively. For the behavioral level, the participants’ confidence as EMI instructors was asked at end of stages 1 and 3. Application of the knowledge and skills gained throughout the training was observed by the EMI lecture shown in stage 3 of training.

## Statistic methods

All questionnaires were conducted through ‘Survey Monkey online (www.sojump.com). Data were expressed as percentages and mean ± standard deviation and were analyzed with independent samples t-test for the question items. Probability value p < 0.05 (two-tailed analysis) tests were considered statistically significant. All statistical analyses were performed using the Statistical software SPSS 25.0 (IBM Corporation).

This study was approved by the Kunming Medical University Research Ethics Committee.

## Results

### Participant characteristics

The baseline survey showed: the mean age of 84 participants was 36 ± 8.5 years. There were 76.19% of participants are female. More than 90% of them were MA or MD of education level, and 72.62% of them did not have EMI teaching experience( Table 1).

**Table 1:** Characteristics of participants

Items	number	percentage
Gender		
male	20	23.81
female	64	76.19
Age		
under 25	3	3.57
26–30	25	29.76
31–40	38	45.24
41–50	17	20.24
51 or over	1	1.19
Qualifications		
Doctorate(e.g.MD/PHD)	34	40.48
Master’s degree(e.g. MA)	42	50.00
University degree (e.g.BA/BEd)	8	9.52
Other (please specify)	0	0.00
EMI teaching experience		
None	61	72.62
0-3years	23	27.38
4years or more	0	0.00

### Participants’ overall reactions

All 84 participants filled in a feedback questionnaire to assess the reaction level at the end of the whole training. All participants were highly satisfied with every aspect listed in the satisfaction survey. (Table 2). Meanwhile, most participants commented trainers as “professional, excellent, responsible, highly experienced and obtained immediate and useful feedback”, and rated the training model as “very well organized and efficient, and we have an all-new perception and understanding on what teaching is and what the responsibility for students is.“

**Table 2:** Overall evaluation of EMI training on the reaction level

Items	strongly disagree	disagree	neither	agree	strongly agree	total
a)This training event met my expectations	0(0%)	0(0%)	0(0%)	41(48.81%)	43(51.19%)	84
b)I have acquired new knowledge and/or skills from taking part in this training	0(0%)	0(0%)	0(0%)	38(45.24%)	46(54.76%)	84
c)This training is relevant to my current role/job	0(0%)	1(1.19%)	3(3.57%)	47(55.95%)	33(39.29%)	84
d)Overall, this was a high quality training event	0(0%)	0(0%)	0(0%)	37(44.05%)	47(55.95%)	84

### Learning level: awareness raising of EMI teaching techniques

All participants completed the survey about “How aware are you now of how to apply the 10 learner-centered techniques for your EMI instruction?“ The survey results showed that participants had higher scores on awareness of EMI teaching techniques after stage 3 compared to after stage 1, and were found to be statistically significant (t = 7.122, P < 0.001). (Table 3) Of note, through reflecting on classroom practice of stage 2 and follow-up training of stage3, the scores of three skills were outstanding increased that scored low at stage1: “Modifying classroom interactions to enhance understanding, Eliciting student ideas through effective questioning and setting group tasks and reducing instructor talk time.“ Moreover, when participants were asked about the most beneficial activities they did during the course, most responses were “planning lessons, group tasks, interactive skills, and eliciting students”.

**Table 3:** Mean score on awareness of 10 EMI teaching techniques

Items	after stage1	after stage3	*p*
	Mean ±	Mean ± SD	Exact sign [2-sided]
a) Modifying language to clearly explain key learning points	3.18 ± 0.83	3.48 ± 0.83	< 0.05
b) Modifying classroom interaction to enhance understanding	3.00 ± 0.78	3.54 ± 1.26	< 0.01
c) Setting up group tasks and reducing tutor talking time	3.32 ± 0.99	3.67 ± 0.76	< 0.01
d) Revising and recycling previously taught content	3.31 ± 1.15	3.53 ± 1.36	< 0.05
e) Eliciting student ideas through effective questioning	3.12 ± 0.52	3.46 ± 0.74	< 0.01
f) Planning lectures to be interactive	3.28 ± 1.25	3.55 ± 1.14	< 0.05
g) oral feedback strategy	3.06 ± 0.61	3.47 ± 1.26	< 0.01
h) Checking understanding	3.31 ± 0.98	3.66 ± 1.17	< 0.05
i) The role of homework	3.38 ± 0.94	3.59 ± 1.10	< 0.05
j) Appreciate the value of self-reflection	3.24 ± 1.06	3.66 ± 0.99	< 0.01

*t = 7.122, P < 0.001*

### Behavior level: confidence as an EMI lecturer improved

**Fig. 2:**
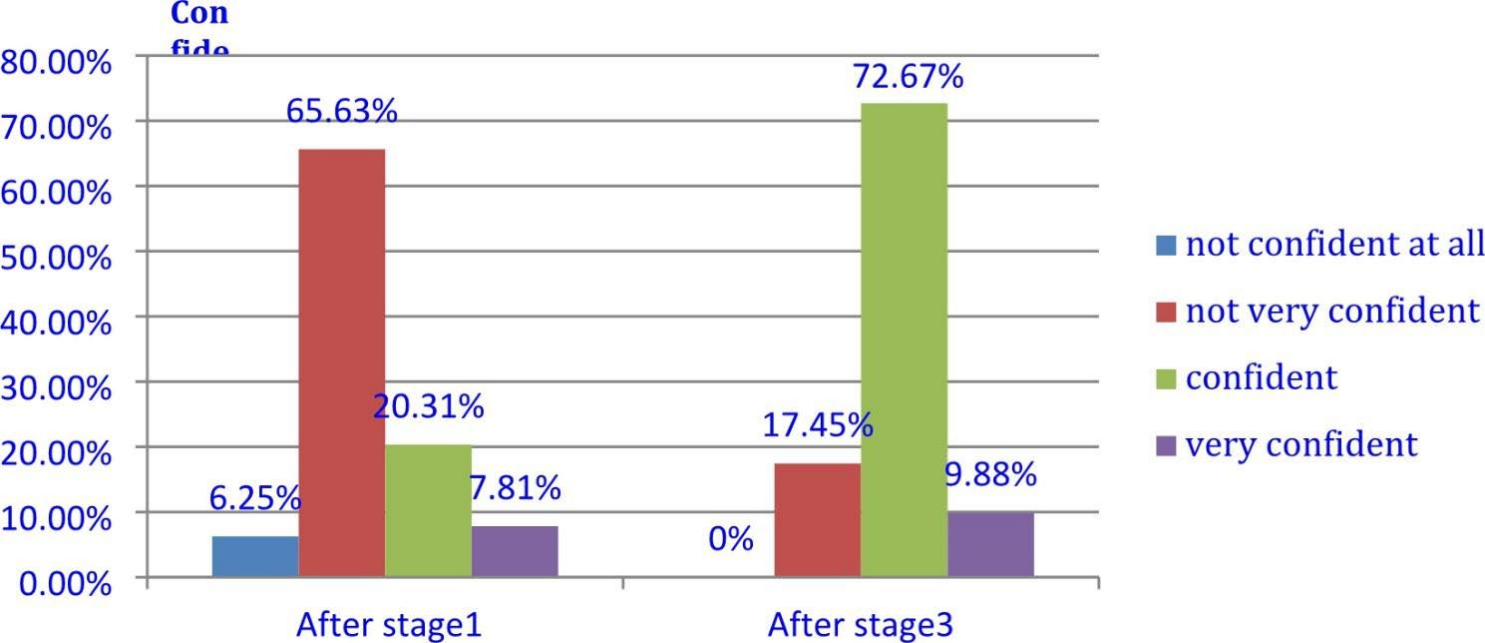
Percentage of participant’s confidence as an EMI instructor

All the participants considered that “as a result of the training, the confidence of themselves as an EMI instructor has grown.“ It increased from 28.12%(20.31% confident and 7.81% very confident) after stage 1 to 82.55%(72.67% confident and 9.88% very confident) after stage 3 (p < 0.001). (Fig. 2) They listed the major improved areas: “Interact with students, assigning group works, controlling the class.“ However, trainers commented that content-based language and functional language in the classroom still require significant continued faculty development to improve instructors’ confidence in using English accurately and fluently in the classroom.

### Behavior level: application of EMI skills improved

Observing trainee’s shows of EMI lecture in stage 3, trainers commented “Teachers are applying EMI teaching skills, such as modifying classroom interactions to enhance understanding, stimulating students’ ideas through effective questioning, and verbal feedback strategies, but they need more consolidation”, and “I see the teachers in action and I think they have more hope for controlling the learning process after this training”, and “The observations and feedback to EMI lectures turned out to be useful”. Feedback from participants more frequently were “we got real learning gains from observing peer’s EMI lectures and received timely feedback from trainers was the very useful for improvement of teaching skills, and practicing and seeing what is going on was most beneficial activities. Moreover, we would like to make some commitment to implement learner-centered learning and would do more practice to sharpen our EMI skills.“

## Discussion

The EMI training project was designed to improve the lecturer’s competence and confidence in EMI instruction and “productivity” when returning to the classroom after the training. In this study, data from three sessions of EMI training were aggregated to evaluate the effectiveness of EIM training based on the 1–3 levels of the Kirkpatrick Evaluation Model. From the results of the satisfaction survey, On the reaction level, 100% of participants had overall high satisfaction with the EMI training project. There was agreement that acquired new knowledge and skills from taking part in this training and met their expectations. The reason was supported by our supplementary open-ended questions and feedback that provide insights into trainees’ perceptions of the trainer and training model of the EMI project. Participants expressed that “trainers’ quality of professionalism, excellence, responsibility, and experience kept them engaged from start to finish”, and “this training allowed us to know exactly what pedagogical (teaching) techniques is, and what teacher’s responsibility is. We learned that teaching is a profession. “

On the learning level of Kirkpatrick, participants’ awareness of 10 learner-centered skills of EMI teaching was increased significantly through two stages of intensive training. It showed that the intensive training of the EMI project was effective and essential for catch-up knowledge and intensive learning for trainees [16]. This result is mainly due to that the learner-centered active learning techniques are the main focus of EMI training that enables motivated trainees as learner role are more focused during learning to experience the learner-centered learning in depth through frequent teacher-learner interactions and peer interactions, as lecturer role to apply the strategies gained from training in their live classroom. This not only enhances the learning experience of learners but also improves teaching skills by practicing. Consequently, trainees’ awareness of EMI teaching skills has changed and also contributed to the shift from traditional lecture-based learning(LBL) to learner-centered learning(LCL).

Furthermore, according to Kirkpatrick learning (level 2) should lead to behavioral changes (level 3). These changes possibly include unquantifiable outcomes such as improved morale and empowerment [17]. This study’s results showed that the participant’s confidence(unquantifiable outcomes) as an EMI instructor at the end of the whole training had substantially increased. This lets us believe that EMI training effectively impacted it. The possible reason is that confidence as an EMI instructor is a crucial factor for the effectiveness of MBBS courses, especially for non-native lecturers of English. Thus, improving trainees’ confidence is another focus of EMI training. In this study, facing most trainees did not have experience of study abroad, and speaking and listening in English wasn’t enough fluency, trainers created a learner-centered learning(LCL) atmosphere that encouraged trainees to use English as much as possible, and enabled every trainee responsible for their learning activity to become active learners to increase their confidence [18].

Although behavior change is challenging and requires more time. But trainees’ behaviors have significantly changed at the end of training. The possible reason is that based on learning by doing, knowledge is translated into experience [19], a practice-based approach was used in this training to enhance participants’ practice and reduce the gap between theory and practice. Particularly, practicing in the live classroom on stage 2 and the EMI lecture show on stage3, greatly contributed trainees to awareness and practice of EMI teaching techniques. In addition, trainees expressed that they would like to make some changes to teaching patterns to sharpen their EMI skills. This also proved that “behavioral level” is a test of learners’ attitudes toward the usefulness of information and knowledge acquired in a given course [20]. Moreover, due to teachers’ English language skills and pedagogy are mainly factors affecting the academic success of international medical students at Chinese universities [21]. To strengthen the lecturers’ ability of the content-based language and functional language should be focused on continuing professional development (CPD)in the future.

From our analyses, it is known that the MEI training project was successful and the effectiveness must extend beyond the achievement of the training learning outcomes [22]. The evaluation results will help educators and policymakers understand the effectiveness and relevance of the training program, correspondingly how to improve it in the future [23].

## Limitations

Our study retained some limitations. First, the provided data are primarily of local interest. The study was based on purposive sampling which only included selected participants from a single university. Thus, limiting the generalisability of the current findings to EMI training programs in other national or international universities. Second, the regular follow-up and the difficulty of applying objective measures to record the improvement in the live classroom during stage 2 and relying on self-reported behavior, might induce reporting bias in the results of level 3 of Kirkpatrick’s evaluation model. Thus, Future follow-up assessments of the performance of those trainees during their lecturing regularly conducted by MBBS students and peers may provide strong evidence of the fourth level of the overall impact of training on organizational performance. Otherwise, further studies may aim to make comparisons between medical universities and produce more generalizable results.

## Conclusion

According to the analytic results, the EMI training has had a favorable impact on all three aspects of the Kirkpatrick model for continuous teacher development assessment. All participants are satisfied with this training project, indicating that it has been effective in strengthening the lecturer’s capacity and learner-centered pedagogy and has the potential to positively impact the teaching quality of MBBS education.

## Electronic supplementary material

Below is the link to the electronic supplementary material.

Supplementary Material 1

Supplementary Material 2

## Data Availability

The datasets analysed during the current study are available from the corresponding author on reasonable request.

## Abbreviations

EMI: English as a Media of Instruction
CPD: Continuing Professional Development
KMU: Kunming Medical University
MBBS: Bachelor of Medicine and Bachelor of Surgery
ATE: Academic Teaching Excellence
LBL: Lecture-Based Learning
LCL: Learner-Centered Learning.
