# Supplementary material for: Assessment of a training project of English as a media of instruction(EMI) using Kirkpatrick model
Source: BMC Med Educ. 2023 Apr 20;23:271. doi: 10.1186/s12909-023-04204-5 (PMC10120192; doi:10.1186/s12909-023-04204-5)
Supplement: Supplementary file 1 — Supplementary Material 1 [file 12909_2023_4204_MOESM1_ESM.docx]

**Kunming Medical University EMI Teacher Development Project**

**End of Whole Training Survey:**The satisfaction questionnaire for EMI training project

Q1. Please tick one box to indicate the extent to which you agree or disagree with

each of the following statements.

| Item | strongly disagree | disagree | neither | agree | strongly agree |
| --- | --- | --- | --- | --- | --- |
| 1.This training event met my expectations |  |  |  |  |  |
| 1. I have acquired new knowledge and/or skills from taking part in this training |  |  |  |  |  |
| 3.This training is relevant to my current role/job |  |  |  |  |  |
| 4.Overall, this was a high quality training event |  |  |  |  |  |

Q2. Do you have any comments about your trainer?

Q3. Do you have any comments about how trainer managed this training?
