# Supplementary material for: Assessment of a training project of English as a media of instruction(EMI) using Kirkpatrick model
Source: BMC Med Educ. 2023 Apr 20;23:271. doi: 10.1186/s12909-023-04204-5 (PMC10120192; doi:10.1186/s12909-023-04204-5)
Supplement: Supplementary file 2 — Supplementary Material 2 [file 12909_2023_4204_MOESM2_ESM.docx]

**Kunming Medical University EMI Teacher Development Project**

**End of 5-day Intensive Training (Stage 1)and Follow-up Training(Stage 3) Survey**

Q1. After the stage1/ stage 3,“ how aware are you now of how to apply the following techniques for your EMI teaching?”

| item | totally unaware | unaware | neither | aware | totally aware |
| --- | --- | --- | --- | --- | --- |
| 1. Modifying language to clearly explain key learning points | 1 | 2 | 3 | 4 | 5 |
| 1. Modifying classroom interaction to enhance understanding | 1 | 2 | 3 | 4 | 5 |
| 3.Setting up group tasks and reducing tutor talking time | 1 | 2 | 3 | 4 | 5 |
| 4.Revising and recycling previously  taught content | 1 | 2 | 3 | 4 | 5 |
| 1. Eliciting student ideas through   effective questioning | 1 | 2 | 3 | 4 | 5 |
| 1. Planning lectures to be interactive | 1 | 2 | 3 | 4 | 5 |
| 7.Oral feedback strategy | 1 | 2 | 3 | 4 | 5 |
| 8.Checking understanding | 1 | 2 | 3 | 4 | 5 |
| 9.The role of homework | 1 | 2 | 3 | 4 | 5 |
| 10. Appreciate the value of self-reflection | 1 | 2 | 3 | 4 | 5 |

Q2. Which activity did you find the most beneficial during the training?

Q3. As a result of the stage 1/ stage 3 training, do you feel your confidence as an EMI lecturer have improved? If,‘YES’, please list the specific areas; if ‘NO’, please state the reason.
